# Improved neutral lipid production from *Tetradesmus obliquus* through fed-batch mixotrophic cultivation at high pH using potato peel hydrolysate

**DOI:** 10.1038/s41598-026-36418-0

**Published:** 2026-02-02

**Authors:** Mohamed Gomaa, Abdel Kareem S. H. Mohamed, Ahmed Mohamed Youssef, Abdel-Rahim A. El-Shanawany

**Affiliations:** 1 Botany & Microbiology Department, Faculty of Science, Assiut University, Assiut, 71516 Egypt; 2 Department of Botany and Microbiology, Faculty of Science, Al-Azhar University, Assiut, 71524 Egypt

**Keywords:** Potato peel hydrolysate, *Tetradesmus obliquus*, Mixotrophic fed-batch cultivation, Alkaline condition, Neutral lipid, Biodiesel properties, Biotechnology, Environmental sciences, Microbiology, Plant sciences

## Abstract

This study investigated the use of potato peel hydrolysate (PPH), obtained through fungal fermentation, as a low-cost organic carbon source to promote the growth and lipid accumulation of *Tetradesmus obliquus* under initial alkaline conditions (pH 11.0). Mixotrophic growth was investigated by incorporating different volumes of PPH to the culture every two days, resulting in final reducing sugar concentrations of 0.01, 0.02, and 0.03 mg mL^− 1^. The mixotrophic fed-batch cultivation (0.02 mg mL^− 1^ PPH) significantly enhanced microalgal biomass and neutral lipid (NL) productivity, reaching 62.73 and 18.70 mg L^− 1^ day^− 1^, respectively, which were 1.8 and 2.5 times higher than the autotrophic control. Moreover, the mixotrophic fed-batch system was evaluated under various nutrient conditions. Low nitrogen or sulfur deprivation notably boosted NL productivity to 20.90 and 22.61 mg L^− 1^ day^− 1^, respectively. The lipids produced under nutrient-limited mixotrophic fed-batch conditions at pH 11.0 were rich in monounsaturated fatty acids (77.49–80.79%) and saturated fatty acids (15.39–19.23%), with the remaining portion comprising polyunsaturated fatty acids. Additionally, various biodiesel properties were assessed, and the results met international standards. These findings suggest that mixotrophic fed-batch cultivation under extreme alkaline conditions can enhance microalgal productivity and promote cost-effective biofuel production.

## Introduction

 The cultivation of microalgae, diverse organisms with a wide array of unique characteristics, has garnered increasing attention in recent years for biodiesel production. Microalgae are known for their rapid growth rates, high photosynthetic efficiency, and the advantage of not require arable land for cultivation^1^. Certain species can accumulate substantial amounts of neutral lipids, mainly in the form of triacylglycerols, which can be readily converted into alkyl esters for biodiesel and other energy sources^2^. The growth patterns and metabolite production of microalgae are highly influenced by the composition of the culture medium (e.g., nitrogen, phosphorus, iron, and sulfur sources and concentrations) as well as environmental conditions such as temperature, pH, and light intensity^3^.

Microalgae can be cultivated under various trophic modes: photoautotrophically (using light as an energy source and CO_2_ as a carbon source), heterotrophically (utilizing organic compounds as both carbon and energy sources in the dark), and mixotrophically (a combination of autotrophy and heterotrophy)^4^. The main drawbacks of autotrophy are the high costs of CO_2_ supply and the relatively low biomass and lipid productivities. Heterotrophic and mixotrophic cultivation can enhance lipid productivity compared to autotrophy, but these methods are hindered by the high costs of organic carbon sources and susceptibility to microbial contamination. Among these, mixotrophic cultivation is often preferred, as it yields greater microalgal biomass than both autotrophy and heterotrophy^5^.

The use of low-cost natural waste materials to stimulate microalgal growth and lipid productivity has been identified as an effective strategy to reduce the costs associated with mixotrophic cultivation^6^. For instance, the use of agri-food wastes such as fruit residues, potato peels, wheat bran, and sugarcane molasses have been reported to enhance microalgal growth and lipid accumulation, thereby supporting circular economy strategies^7–12^. Potato peel waste is primarily generated by the food processing industry, especially in the production of potato-based products like chips, fries, and dehydrated potatoes. The peeling process can result in waste ranging from 15% to 40% of the initial product mass, depending on the peeling method used. A total of 35.5 million tonnes of potato waste are generated globally^13^. Potato peels, often considered a useless waste, pose significant environmental challenges due to their rapid microbial spoilage when discarded without proper treatment. However, they are rich in nutrients and organic compounds, such as cellulose, hemicellulose, starch, and lignin, making them suitable for mixotrophic cultivation of various microalgae and cyanobacteria^14^. Proper pretreatment is essential to break down these macromolecules into fermentable sugars that can be utilized by microalgal cells during growth. Despite their potential, there have been no attempts to use potato peels for mixotrophic microalgae cultivation under alkaline conditions, particularly with varying concentrations of nitrogen, phosphorus, and sulfur.

Cultivating microalgae under alkaline conditions can help to prevent or mitigate microbial and predator contamination in mixotrophic cultures, which increases stability and minimizes culture crashes^15^. Alkaline environments also promote higher CO_2_ bio-fixation rates and increase CO_2_ scavenging from the atmosphere, supporting enhanced microalgal growth^16^. For instance, CO_2_ solubility from the air (0.04 vol% CO_2_) in water increases with pH: 2.0 × 10^− 5^ mol, 8.0 × 10^− 5^ mol, 7.0 × 10^− 4^ mol, and 7.0 × 10^− 3^ mol CO_2_ per liter of water at pH values of 6.0, 7.0, 8.0, and 9.0, respectively^17^. Alkaliphilic algae, which thrive in environments with high pH values (pH > 9), are considered promising candidates for large-scale cultivation and biofuel production^15,16^. Due to their ability to survive and flourish at elevated pH levels, these algae offer a promising avenue for sustainable, large-scale biofuel generation^15,16^.

The aim of this study was to evaluate an ecologically sustainable process for utilizing potato peels as a cost-effective organic carbon source in the fed-batch mixotrophic cultivation of *Tetradesmus obliquus* under alkaline conditions. Fungal fermentation was employed as a simple and low-cost method for pretreating potato peels. The effects of varying volumes of potato peel hydrolysate, nitrogen, phosphorus, and sulfur in the culture medium were assessed in relation to algal biomass productivity and the production of phospholipids, glycolipids, and neutral lipids. Additionally, the fatty acid methyl ester profile was analyzed, and potential biodiesel properties were calculated in accordance with international standards.

## Materials and methods

### Collection of potato peels and pretreatment

Potato peels from Irish potato (*Solanum tuberosum*) were collected from local restaurants in the El-Sharqia region of Egypt. They were washed thoroughly with water to remove any undesirable particles and dried in a hot-air oven at 45 °C until reaching a constant weight, then crushed using a mortar and pestle. The resulting powdered samples were stored in airtight bags at room temperature until further use.

The pretreatment of the powdered potato peels was carried out using semi-solid-state fermentation with the filamentous fungus *Aspergillus niger*. For this, the powdered peels (70 g) were suspended in distilled water (700 mL) and autoclaved at 121 °C for 20 min under 1.5 bar pressure. The samples were then inoculated with an *A. niger* spore suspension (1 × 10⁷ cfu mL^− 1^) and fermented at 27 °C for 4 days under continuous shaking at 120 rpm. After fermentation, the medium was filtered to remove residual biomass and fungal mycelia. The concentration of reducing sugars in the PPH was determined spectrophotometrically using the dinitrosalicylic acid (DNS) method^18^, using glucose as a standard. The concentrations of total sugars, proteins, and lipids in the PPH were estimated using UV-H_2_SO_4_ method^19^, Lowry method^20^, and phosphovanillin method^21^. The concentrations of phosphate, sulphate, and nitrate were estimated spectrophotometrically using standard methods^22–24^.

### Microalgal species and growth conditions

*Tetradesmus obliquus* was isolated from a water sample collected from El-Ibrahimiya canal at Assiut, Egypt. The identification of the microalga followed keys and descriptions of Bellinger and Sigee^25^. The microalgal growth was carried out using an alkaline medium (AM) of the following composition (g L^–1^): NaNO_3_, 0.25; NH_4_Cl, 0.05; MgSO_4_.7H_2_O, 0.075; CaCL_2_.2H_2_O, 0.025; NaCl, 0.025; Ferric ammonium citrate, 0.01; K_2_HPO_4_, 0.25; Na_2_CO_3_, 0.25; H_3_BO_3_, 2.4 × 10^− 3^; MnCl_2_.4H_2_O, 1.0 × 10^− 3^; ZnCl_2_, 0.08 × 10^− 3^; CuCl_2_.2H_2_O, 0.06 × 10^− 3^; NaMoO_4_.2H_2_O, 0.06 × 10^− 3^; CoCl_2_.6H_2_O, 0.06 × 10^− 3^; NiCl_2_.6H_2_O, 0.04 × 10^− 3^; KBr, 0.04 × 10^− 3^ in 1.0 L of distilled water (pH 11.0). The inoculum of *T. obliquus* was prepared by cultivation in 250 mL of sterile alkaline medium (pH 11.0) in a 500 mL glass bottles under continuous illumination (48.4 µmol m^− 2^s^− 1^) at 25 ± 2 °C for 7 days. The culture was aerated with sterile air, and cells in the exponential phase were harvested and used as the inoculum for subsequent experiments.

The growth of the microalga was tested at different initial pH values (7–11) to evaluate the effect of alkaline conditions on the algal growth and biomass productivity. In this experiment the initial pH of the AM was adjusted prior to autoclaving, and the initial cell concentration was set to 0.1 units of optical density at 750 nm using a vis- spectrophotometer (JENWAY 7315).

### Fed-batch mixotrophic cultivation

The PPH was used as an organic carbon source for the mixotrophic cultivation of *Tetradesmus obliquus*. Algal cells were harvested by centrifugation (4800 g, 15 min) and used to inoculate 200 mL of sterilized AM medium in 250 mL conical flasks (initial pH 11), resulting in a final optical density (OD) of 0.1 at 750 nm, equivalent to 0.08 ± 0.005 g L^− 1^. Mixotrophic growth was conducted using a fed-batch cultivation method, with different volumes of PPH added at regular intervals. Aliquots of 1, 2, and 3 mL of PPH, containing 2.18 mg mL^− 1^ of reducing sugars, were fed into the microalgal medium every two days, achieving final reducing sugar concentrations of 0.01, 0.02, and 0.03 mg mL^− 1^, respectively. The mixotrophic growth proceeded for 10 days under these conditions and was compared with an autotrophic culture, prepared similarly but without the addition of PPH.

### Fed-batch mixotrophic cultivation under different nutrient concentrations

A second experiment was also conducted to evaluate the mixotrophic growth under nutrient limited and deprived conditions as listed in Table 1. All the treatments were cultivated mixotrophically by feeding the culture medium with 2 mL of PPH every 2-days. Microalgal growth was proceeded under the aforementioned growth conditions.

**Table 1:** Different nutrient limited and deprived conditions for the fed-batch mixotrophic growth of *T. obliquus* using 2 mL potato Peel hydrolysate at 2-days interval.

Code	Name	Treatment conditions
**T1**	Control	Alkaline medium (AM)
**T2**	Nitrogen deprivation	The AM lacked NaNO_3_ and NH_4_Cl
**T3**	Low nitrogen	The AM lacked NaNO_3_ but contained 0.05 g L^–1^ NH_4_Cl
**T4**	Moderate nitrogen	The AM contained 0.1 g L^–1^ NaNO_3_ and 0.05 g L^–1^ NH_4_Cl
**T5**	Phosphate deprivation	The AM lacked K_2_HPO_4_
**T6**	Low phosphate	The AM contained 0.1 g L^–1^ K_2_HPO_4_
**T7**	Sulphate deprivation	The AM lacked MgSO_4_.7H_2_O
**T8**	Low sulphate	The AM contained 0.02 g L^–1^ MgSO_4_.7H_2_O

### Evaluation of cell growth

An aliquot of microalgal culture was taken at regular intervals, and its optical density (OD) was measured at 750 nm in order to calculate the cell density. For this purpose, a series of microalgal cultures with varied OD values were collected by centrifugation (4000 g, 15 min), and the pellet was subsequently oven-dried (60 °C)^26^. The OD values were converted into dry cell weight (DCW) (mg L^− 1^) using a standard curve. Using the following formula^6^, the algal biomass productivity (BP) was determined:

1 $$\:BP\left(\mathrm{m}\mathrm{g}\:{L}^{-1}{day}^{-1}\right)\:=\frac{({X}_{t}-{X}_{0})}{\varDelta\:t}$$

where *X*_*t*_ is the DCW at the end of experiment (mg L^− 1^). *X*_*0*_ is the initial DCW (mg L^− 1^), and Δ*t* is the total duration of fed batch cultivation (day).

### Lipid analysis

#### Determination of total lipids (TL)

Centrifugation was utilized to concentrate the microalgal cells, and the pellet was then resuspended in a predetermined amount of distilled water. After adding 2 mL of concentrated H_2_SO_4_ to 200 µL of the concentrated algal cells, the mixture was heated for 10 min at 100 °C in a water bath. Each tube received 5 mL of the phosphovanillin reagent after cooling^21,27^. After 15 min, the absorbance was measured spectrophotometrically at 530 nm. Sunflower oil was utilized as a standard^21,27^.

#### Estimation of Polar and non-polar lipids

The extraction of total lipids (TL) from the wet microalgal biomass was performed using chloroform: methanol (2:1 v/v) with shaking for 48 h, followed by centrifugation to remove the residual cells. The extracts were evaporated at 60 °C and 0.75 mL of HCl (3 M) was introduced to each tube for lipid hydrolysis for 2 h at 100 °C, then the volume was completed to 2 mL with deionized water. The galactose released from glycolipids (GL) in the lipid hydrolysate was determined by phenol sulfuric acid method^28^ at 490 nm using galactose as a standard. The concentration of galactose was multiplied by a factor (100/35 to) for conversion into GL^29^. On the other hand, the released phosphate from phospholipids (PhL) was estimated spectrophotometrically using molybdenum blue method^22^ using K_2_HPO_4_ as standard. PhL was computed by multiplying P concentration by 25^29^. Polar lipids (PL) were calculated as PL = GL + PhL, while non-polar lipids (NL) were estimated as NL = TL – PL.

### Determination of fatty acid methyl esters (FAME)

The total lipids from the investigated microalga under optimum growth conditions were extracted using chloroform: methanol (2:1) and converted into FAME as described previously^30^. The FAME profile was identified using gas chromatography/mass spectrophotometry (GC/MS) in the Analytical Chemistry Unit in the Chemistry Department, Faculty of Science, Assiut University, Egypt using the method reported previously^31^.

### Biodiesel characteristics

The *FAME* profile of the *T. obliquus*was used for study of the biodiesel characteristics using the following Eqs^32,33^:

2 $$\:Saponification\:value,\:SV=\:\sum\:(560\times\:N)/MW$$

3 $$\:Iodine\:value,\:IV=\sum\:(254\times\:N\times\:D)/MW$$

4 $$\:Cetane\:EquationNumber,\:CN=46.3+5458/SV-(0.225\times\:IV)$$

5 $$\:Degree\:of\:unsaturation,\:DU=\sum\:MUFA+(2\times\:PUFA)$$

6 $$\:Oxidation\:stability,\:OS=-0.0384\times\:DU+7.77\:$$

7 $$\begin{aligned}\:Long-chain\:saturation\:factor,\:LCSF&=\left(0.1\times\:C16:0\right)+\left(0.5\times\:C18:0\right)\\&\quad+\left(1\times\:C20:0\right)+\left(2\times\:C24:0\right)\end{aligned}$$

8 $$\:Cold\:filter\:plugging\:point,\:CFPP=\left(3.1417\times\:LCSF\right)-16.477\:$$

9 $$\:Cloud\:point,\:CP=\left(0.526\times\:C16\right)-4.992\:$$

10 $$\:Pour\:point,\:PP=\left(0.571\times\:C16\right)-12.24$$

11 $$\:Kinematic\:viscosity,\:{ln}{v}_{i}=-12.503+(2.496\times\:{ln}MW)-(0.178\:\times\:N)$$

12 $$\:Density,{\rho\:}_{i}=0.8463+(4.9/MW)+(0.0118\times\:N)$$

13 $$\:Higher\:heating\:value,\:HHV=46.19-(1794/MW)-(0.21\times\:N)$$

14 $$\begin{aligned}\:Flash\:point,\:FP&=205.226+0.083\times\:C16:0-1.723\times\:C18:0-0.5717\times\:C18:1\\&\quad-0.3557\times\:C18:2-0.46\times\:C18:3-0.2287\times\:C22\end{aligned}$$

where *N* is the % of FAME, *D* is the number of double bonds, *MW* is the molecular weight, *MUFA* is the monounsaturated FAME and *PUFA* is the polyunsaturated FAME.

### Statistical analyses and cellular growth modeling

At the 0.05 significance level, an analysis of variance (ANOVA) with post hoc Fisher’s least significant difference (LSD) testing was used to examine the differences between treatment means using GNU PSPP statistical program (v 1.6.2).

The microalgal growth kinetics under different treatments were fitted to the modified logistic model using the following Eqs^34^,

15 $$\:X\left(t\right)={X}_{0}+\:\frac{({X}_{max}-{X}_{0})}{1+exp\:\left\{\left(\frac{4{\mu\:}_{max}}{{X}_{max}-{X}_{0}}\right)\left(\lambda\:-t\right)+2\right\}}$$

where *X(t)*, *X*_*0*_, *X*_*max*_ are the time dependent increase in the microalgal biomass (g L^− 1^), the initial biomass concentration and the maximum biomass concentration, respectively. While *µ*_*max*_ indicates the maximum growth rate (day^− 1^) and *λ* is the lag time (day). The microalgal growth as a function of time was solved by applying the Newton’s method in Microsoft Excel 2016 software by minimizing the value of the root mean square error (RMSE):

16 $$\:RMSE=\:\sqrt{\frac{\sum\:_{t=1}^{N}{({X\left(t\right)}_{calc}-\:{X\left(t\right)}_{exp})}^{2}}{N}}$$

where *X(t)*_*calc*_ and *X(t)*_*exp*_ represent the calculated and the actual microalgal biomass at time *t* and *N* is the number of experimental points.

## Results

### Effect of pH on *T. obliquus* growth, lipid content, and lipid productivity

**Fig. 1:**
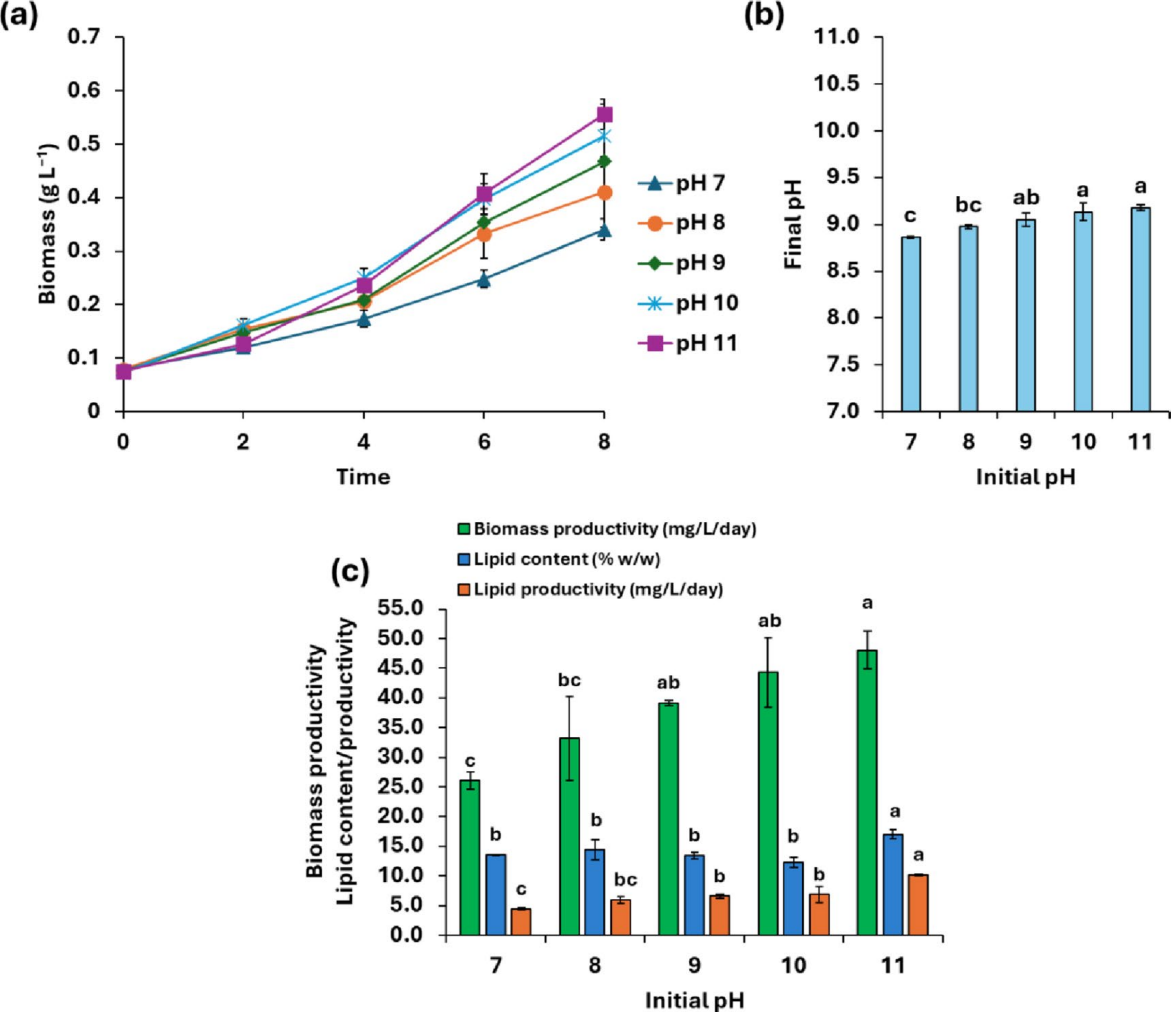
Growth curves (**a**), variations in biomass productivity, lipid content/productivity (**b**), and variation of final culture pH (**c**) of *T. obliquus* under photo-autotrophic conditions at different initial pH values. Values are measured as mean ± standard deviation. Different letters above columns indicate significant differences at P-value < 0.05.

The biomass productivity of *T. obliquus* demonstrated a remarkable increase under alkaline pH conditions (8–11); ranging from 1.3 to 1.8-fold compared to neutral conditions (pH 7, 26.06 mg L^− 1^ day^− 1^) (Fig. 1a, b). Following 10 days of cultivation, the culture medium exhibited an alkaline pH, reaching 8.87 at an initial pH of 7.0, while the final culture pH was 9.14 and 9.18 when the initial pH values were 10 and 11, respectively (Fig. 1b). A significant enhancement in cellular lipid contents was observed at extreme alkaline conditions (pH 11), which increased to 17.00% (w/w) compared to 13.55% (w/w) at pH 7 (Fig. 1c). In contrast, non-significant variations in lipid contents were observed in the pH range 8 − 10 in relation to pH 7. Additionally, lipid productivity also showed a significant promotion to 6.57–10.20 mg L^− 1^ day^− 1^ under alkaline pH (9–11), which was estimated to be 1.5–2.3-fold higher than the control at pH 7 (4.41 mg L^− 1^ day^− 1^) (Fig. 1c).

### Effect of mixotrophic fed-batch cultivation on *T. obliquus* growth and lipid composition

The PPH sample contained reducing sugars (2.18 g L^–1^), lipids (0.18 g L^–1^), proteins (4.76 g L^–1^), total carbohydrates (82.71 g L^–1^), and phenolic compounds (0.04 g L^–1^). The PPH also contained inorganic nutrients such as nitrate (6.34 mg L^–1^), phosphate (0.11 mg L^–1^), and sulphate (0.41 mg L^–1^).

The mixotrophic cultivation of *T. obliquus* using PPH was evaluated at alkaline pH (pH 11) since it significantly enhanced the algal biomass and lipid productivities under autotrophic conditions.

**Fig. 2:**
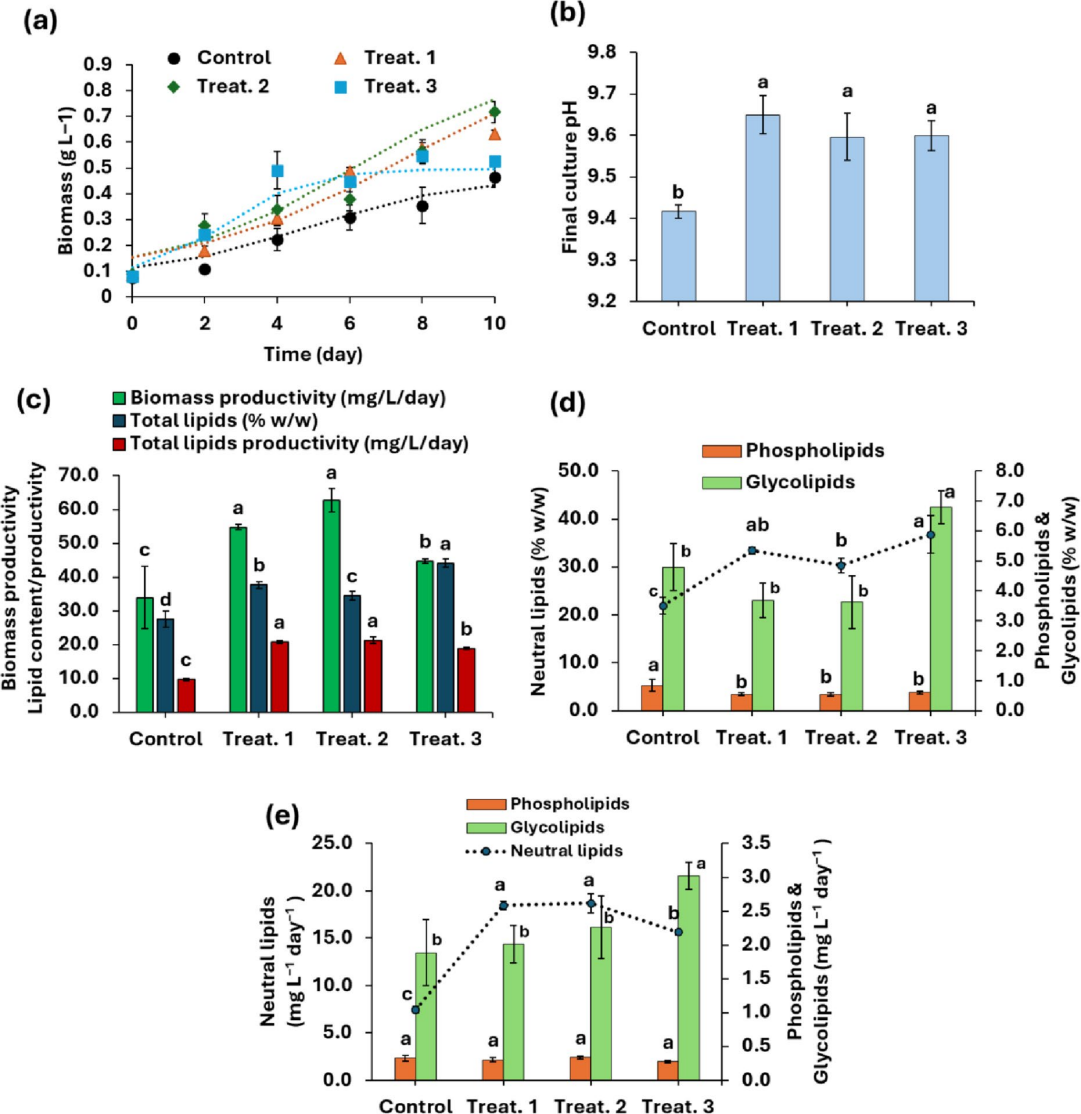
(**a**) Growth curves fitted by modified logistic model (dotted lines), (**b**) variations in final culture pH, (**c**) variation in biomass productivity, total lipids contents/productivity, (**d**) contents of neutral lipids, phospholipids, and glycolipids, and (**e**) productivities of neutral lipids, phospholipids, and glycolipids of *T. obliquus* under mixotrophic fed-batch conditions at initial pH 11 using different concentrations (Treat. 1: 1 mL, Treat. 2: 2 mL, and Treat. 3: 3 mL of potato peel hydrolysate) in relation to autotrophic control. Values are measured as mean ± standard deviation. Different letters above columns indicate significant differences at P-value < 0.05.

Figure 2a. depicts the growth curves of the microalga under fed-batch mixotrophic conditions at different volumes of PPH. The final culture pH after 10 days of growth in the autotrophic control was 9.42 ± 0.015, while in the mixotrophic fed-batch treatments, the pH values were 9.65 ± 0.046, 9.60 ± 0.057, and 9.60 ± 0.036 using 1, 2, and 3 mL of PPH, respectively (Fig. 2b).

The results indicated significant enhancements in the *T. obliquus* growth and biomass productivity by incorporating 1 and 2 mL of PPH under fed-batch conditions. Under these treatments the biomass productivity exhibited ~ 1.6–1.8-fold increase (54.75 ± 0.80–62.73 ± 3.48 mg L^− 1^ day^− 1^) in relation to the autotrophic control (33.92 ± 9.29) (Fig. 2c). Additionally, the incorporation of 3 mL of PPH showed significant promotion in the algal growth and biomass production, but the enhancement was only 1.3-fold higher than the control treatment (Fig. 2c).

The microalgal growth was fitted with the modified logistic model, which indicated a reasonable agreement with the experimental values as indicated by high coefficient of determination values and low RMSE values (Table 2). The results indicated a significant enhancement of maximum growth rates (µ_max_) of *T. obliquus* under mixotrophic conditions compared to autotrophic control, and the highest values were obtained using 2 and 3 mL PPH (Table 2). However, these treatments exhibited an increase in the lag-time (λ), since the microalgal cells require a long period to adapt themselves to these mixotrophic conditions.

**Table 2:** Variations of maximum growth rate (µ_max_) and lag time of *T. obliquus* growth under mixotrophic fed-batch conditions at initial pH 11 using different concentrations (1, 2, and 3 mL of potato Peel hydrolysate) in relation to the autotrophic control.

Treatments	µ_max_	Lag time	*R* ^2^	RMSE
**Control**	0.05 ± 0.01^c^	0.86 ± 0.71^b^	0.99	0.02
**1 mL**	0.08 ± 0.002^b^	1.19 ± 0.05^b^	0.98	0.05
**2 mL**	0.11 ± 0.009^a^	1.82 ± 0.69^ab^	0.98	0.05
**3 mL**	0.12 ± 0.008^a^	2.40 ± 0.38^a^	0.75	0.19

Values are measured as mean ± standard deviation.

Different superscript letters indicate significant differences at p < 0.05.

R^2^: coefficient of determination.

RMSE: root mean square error.

The contents of different lipid classes varied significantly between treatments. Phospholipid levels were reduced by nearly 30% in the mixotrophic treatments compared to the autotrophic control (Fig. 2d). In contrast, glycolipid levels increased by approximately 40% with the addition of 3 mL of PPH under fed-batch conditions compared to the control (Fig. 2d). Despite these changes, the overall productivity of polar lipids in the mixotrophic treatments showed no significant variation compared to autotrophic control (Fig. 2e).

On the other hand, *T. obliquus* cells accumulated more neutral lipids (NL) under mixotrophic conditions, with their content ranging from 30.29% to 36.72% (w/w) compared to 21.90% (w/w) in the autotrophic control (Fig. 2d). These increases were 1.38 to 1.68 times higher than the control. Additionally, NL productivity was significantly enhanced under mixotrophic conditions, reaching a maximum of approximately 18.5 mg L^− 1^ day^− 1^ with the addition of either 1 mL or 2 mL of PPH. This value was about 2.5 times higher than that of the autotrophic control (Fig. 2e).

Analysis of the total lipid content indicated a significant increase under fed-batch mixotrophic cultivation with varying concentrations of PPH compared to the autotrophic control (Fig. 2c). In the control treatment, total lipids accounted for 27.54% (w/w), whereas supplementation with 1, 2, and 3 mL of PPH resulted in significant increases to 37.67%, 34.47%, and 44.12% (w/w), respectively (Fig. 2c). Similarly, the total lipid productivity increased significantly to 20.77, 21.30, and 18.92 mg L^− 1^ day^− 1^ with the addition of 1, 2, and 3 mL PPH, respectively, representing approximately a twofold increase compared to the control (9.66 mg L^− 1^ day^− 1^) (Fig. 2c).

### Effect of different nutrients on *T. obliquus* growth and lipid composition under mixotrophic fed-batch cultivation

**Fig. 3:**
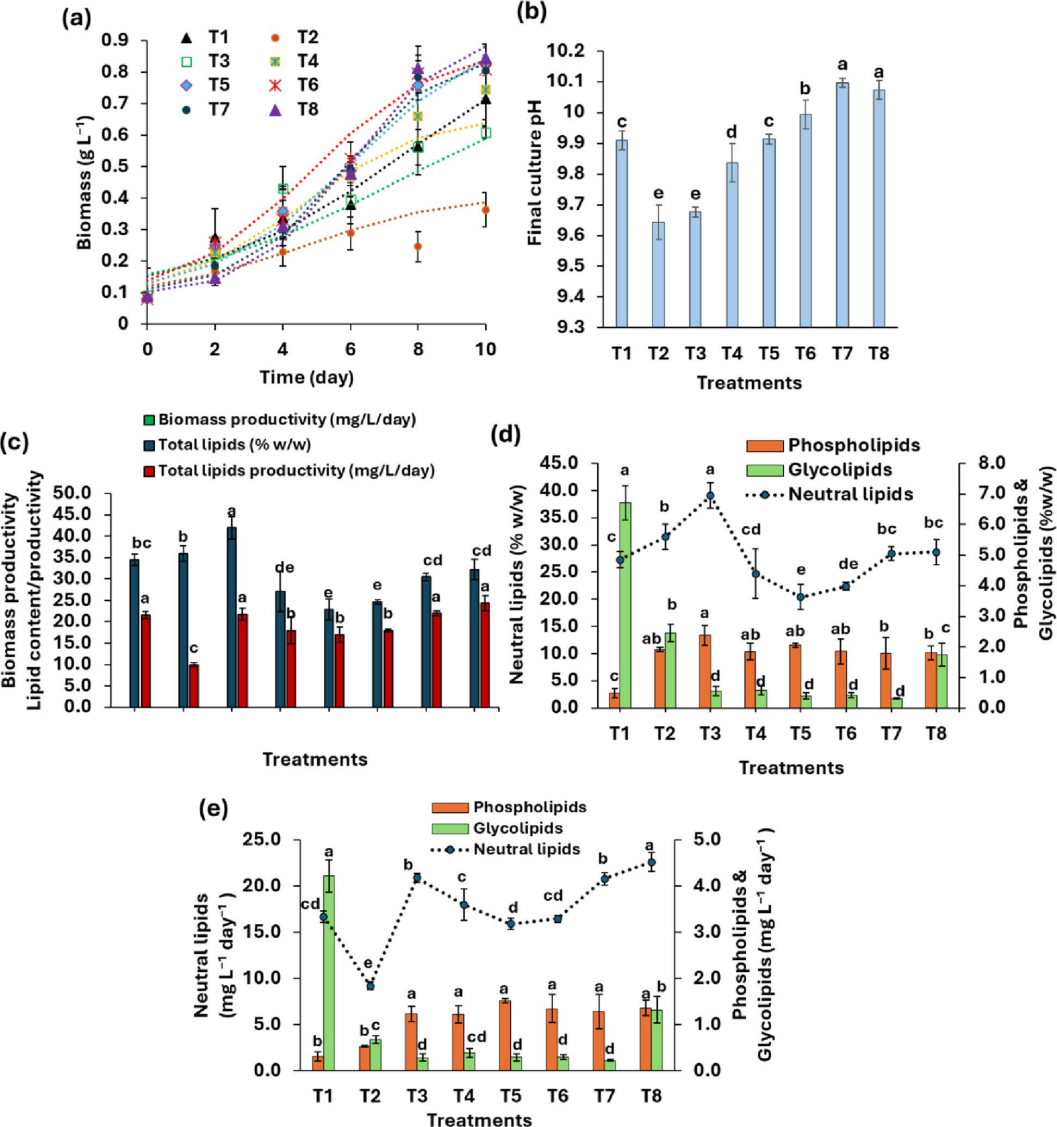
(**a**) Growth curves fitted by modified logistic model (dotted lines), (**b**) variations in final culture pH, (**c**) variation in biomass productivity, total lipids contents/productivity, (**d**) contents of neutral lipids, phospholipids, and glycolipids, and (**e**) productivities of neutral lipids, phospholipids, and glycolipids of *T. obliquus* under mixotrophic fed-batch conditions at initial pH 11 using 2 mL of potato peel hydrolysate and different conditions of nutrient availability. T1: Control (alkaline medium (AM)), T2: Nitrogen deprivation (the AM lacked NaNO_3_, and NH_4_Cl), T3: Low nitrogen (the AM lacked NaNO_3_ but contained 0.05 g L^–1^ of NH_4_Cl), T4: Moderate nitrogen (the AM contained 0.1 g L^–1^ of NaNO_3_ and 0.05 g L^–1^ of NH_4_Cl), T5: Phosphate deprivation (the AM lacked K_2_HPO_4_), T6: Low phosphate (the AM contained 0.1 g L^–1^ of K_2_HPO_4_), T7: Sulphate deprivation (the AM lacked MgSO_4_.7H_2_O), T8: Low sulphate (the AM contained 0.02 g L^–1^ of MgSO_4_.7H_2_O). Values are measured as mean ± standard deviation. Different letters above columns indicate significant differences at P-value < 0.05.

Figure 3a. depicts the growth curves of *T. obliquus* under fed-batch mixotrophic conditions using 2 mL of PPH under different nutrient concentrations. The analysis of final culture pH after 10 days of growth indicated that low or deprived sulphate-maintained culture pH at above 10. However, all treatments maintained final pH at above 9.5 (Fig. 3b).

The results presented in Fig. 3a, c indicate a significant increase in the biomass productivity of *T. obliquus* under phosphate- or sulfate-deprived conditions (T5 and T6 for phosphate, T7 and T8 for sulfate) during mixotrophic fed-batch cultivation. Under these treatments, biomass productivity reached 72.05–75.65 mg L^− 1^ day^− 1^, approximately 1.2 times higher than the nutrient-sufficient control (62.73 mg L^− 1^ day^− 1^) (Fig. 3c). Conversely, biomass productivity was negatively affected when nitrogen salts (NH_4_Cl and NaNO_3_) were omitted from the mixotrophic culture (T2). Similarly, low nitrogen conditions (T3: 0.05 g L^− 1^ NH_4_Cl) led to a significant reduction in biomass productivity, whereas it was restored under moderate nitrogen concentrations (T4: 0.1 g L^− 1^ NaNO_3_ and 0.05 g L^− 1^ NH_4_Cl) (Fig. 3c).

These trends corresponded with the treatments’ effects on microalgal growth rates. The modified logistic model showed a significant reduction in µmax values under nitrogen- (T2) and sulfate-deprived (T7) conditions (Table 3). Additionally, the lag time was prolonged under low nitrogen (T3), phosphate-deprived (T5), and low sulfate (T8) conditions (Table 3).

**Table 3:** Variations of maximum growth rate (µ_max_) and lag time of *T. obliquus* at initial pH 11 using 2 mL of potato Peel hydrolysate and different conditions of nutrient availability. T1: control (alkaline medium (AM)), T2: nitrogen deprivation (the AM lacked NaNO_3_, and NH_4_Cl), T3: low nitrogen (the AM lacked NaNO_3_ but contained 0.05 g L^–1^ of NH_4_Cl), T4: moderate nitrogen (the AM contained 0.1 g L^–1^ of NaNO_3_ and 0.05 g L^–1^ of NH_4_Cl), T5: phosphate deprivation (the AM lacked K_2_HPO_4_), T6: low phosphate (the AM contained 0.1 g L^–1^ of K_2_HPO_4_), T7: sulphate deprivation (the AM lacked MgSO_4_.7H_2_O), T8: low sulphate (the AM contained 0.02 g L^–1^ of MgSO_4_.7H_2_O).

Treatments	µ_max_	Lag time	*R* ^2^	RMSE
**T1**	0.14 ± 0.003^ab^	2.19 ± 0.70^b^	0.96	0.17
**T2**	0.10 ± 0.003^cd^	1.95 ± 0.35^bc^	0.87	0.19
**T3**	0.14 ± 0.01^ab^	3.63 ± 0.15^a^	0.87	0.14
**T4**	0.12 ± 0.001^bc^	1.50 ± 0.15^bc^	0.95	0.13
**T5**	0.19 ± 0.026^a^	3.42 ± 0.37^a^	0.97	0.08
**T6**	0.15 ± 0.015^ab^	1.04 ± 0.04^c^	0.98	0.14
**T7**	0.07 ± 0.01^d^	0.99 ± 0.65^c^	0.98	0.07
**T8**	0.11 ± 0.04^bc^	3.75 ± 1.19^a^	0.94	0.10

Values are measured as mean ± standard deviation.

Different superscript letters indicate significant differences at *p* < 0.05.

R^2^: coefficient of determination.

RMSE: root mean square error.

The phospholipid content in *T. obliquus* cells cultivated mixotrophically showed a significant increase under nutrient-limited or deprived conditions compared to the control (Fig. 3c). In contrast, glycolipid content exhibited the opposite trend (Fig. 3c). In the autotrophic control, the contents of phospholipids and glycolipids were 0.49% and 6.71% (w/w), respectively. However, under nitrate-, sulfate-, and phosphate-limited or deprived conditions, phospholipid content significantly increased to 1.78–2.37% (w/w), while glycolipid content significantly decreased to 0.31–2.44% (w/w) (Fig. 3c). Furthermore, there was a notable reduction in overall polar lipid productivity, ranging from 1.4- to 1.77-fold lower than the control.

Conversely, the NL content in the mixotrophically cultivated cells reached 39.08% (w/w) at low nitrate concentrations, which represents a 1.43-fold increase compared to the normal autotrophic condition (27.25% w/w) (Fig. 3c). However, there were no significant effects on NL content under moderate or deprived nitrogen conditions (Fig. 3c). Similarly, the removal or reduction of sulfate in the mixotrophic culture did not significantly impact the NL content of *T. obliquus* compared to the control. In contrast, phosphate-limited or deprived conditions significantly decreased NL content to 20.40–22.38% (w/w).

Additionally, NL productivities showed significant enhancement under low nitrate, low sulfate, and sulfate-deprived conditions, reaching 20.90, 22.61, and 20.78 mg L^− 1^ day^− 1^, respectively (Fig. 3d). These values were approximately 1.25 to 1.35 times higher than the control level of 16.69 mg L^− 1^ day^− 1^.

Analysis of total lipid content revealed a significant increase under low-nitrogen conditions (T3) compared to the control (Fig. 3c). In contrast, total lipid productivity showed no significant differences under low-nitrogen (T3), sulfate-deprived (T7), or low-sulfate (T8) conditions relative to the control (Fig. 3c).

A detailed comparison of the biomass and lipid productivities of *T. obliquus* in relation to previous studies in mixotrophic cultivation is presented in Table 4. In this study, mixotrophic cultivation was performed under alkaline conditions using a low concentration of potato peel hydrolysate in a fed-batch mode. The lipid productivity obtained in this study was comparatively higher than those reported for *Scenedesmus obliquus* cultivated using food wastewater^35^, and *Tetraselmis indica* using kinnow peel^36^, but lower than that reported for *Chlorella sorokiniana* using mixed peel extracts^37^.

**Table 4:** Comparison between biomass productivity, lipid content, and lipid productivity between the mixotrophic fed-batch cultivation proposed in the present study and previous studies.

Microalga	Waste pretreatment	Medium	pH	Light intensity	Biomass	Lipid		Ref.
					mgL^− 1^day^− 1^	%w/w	mgL^− 1^day^− 1^	
*Synechococcus elongatus* BDU 10,144	Potato peel waste (PPW) (Ultrasonication)	PPW (10%) in fertilizer seawater medium	9	50 µmol m^− 2^s^− 1^ (continuous)	120.70	-	-	(Chandra & Mallick, 2022)
*Tetraselmis indica*	Kinnow peel (Homogenization)	Peel extract in sewage wastewater	7	94.5 µmol m^− 2^s^− 1^ (16 light: 8 dark h)	54.77	32	17.52	Amit & Kumar Ghosh^36^
*Scenedesmus obliquus*	-	Food wastewater (1%) in Bold’s Basal medium	-	120 µmol m^− 2^s^− 1^ (16 light: 8 dark h)	-	19.7	13.30	Ji et al.^35^
*Chlorella sorokiniana*	Potato, banana, and sweet lime (acid pretreatment followed by enzymatic hydrolysis)	25% mixed waste in water	7	5000 lx (16 light: 8 dark h)	206.00	25.87	53.29	Malakar et al.^37^
*Chlamydomonas* sp. RCC2488 (Malina)	Potato peel waste (acid pretreatment in autoclave followed by enzymatic hydrolysis)	10% v/v PPH in modified f/2 medium	-	120 µmol m^− 2^s^− 1^ (continuous)	39.3	45.00	-	Urme et al.^9^
*Spirulina sp.*	Potato peel waste (acid pretreatment in autoclave followed by enzymatic hydrolysis)	10% v/v PPH in BG-11 medium	7.5	2000 lx (12 dark:12 light h)	59.84	19.87	-	Nguyen^11^
*Tetradesmus obliquus*	Potato peel waste (Fungal fermentation)	1% v/v potato peel hydrolysate every 2 days (synthetic medium)	11	48.4 µmol m^− 2^s^− 1^ (continuous)	75.65	32.24	24.39	**This study**

- Not reported.

### Biodiesel properties

The highest lipid productivity of *T. obliquus* was observed under low nitrate (T3) and sulfate (T8) concentrations; thus, these treatments were analyzed to identify the fatty acid methyl esters (FAME) and their biodiesel characteristics in comparison to the control (T1). The FAME profile indicated higher percentages of monounsaturated fatty acids (MUFAs), which contributed 74.59%, 80.79%, and 77.49% in T1, T3, and T8, respectively (Table 5). The percentages of saturated fatty acids (SFAs) were lower, reaching 25.40%, 15.39%, and 19.23% in T1, T3, and T8, respectively. Furthermore, no polyunsaturated fatty acids (PUFAs) were detected in control, while lower percentages of PUFAs were observed in T3 (3.82%) and T8 (3.27%) (Table 5).

**Table 5:** Percentage of fatty acids of *T. obliquus* under mixotrophic fed-batch treatments using 2 mL potato Peel hydrolysate incorporated every 2 days. Control: growth under nutrient sufficient medium, T3: low nitrogen (the AM lacked NaNO_3_ but contained 0.05 g L^–1^ of NH_4_Cl), T8: low sulphate (the AM contained 0.02 g L^–1^ of MgSO_4_.7H_2_O).

Fatty acids (%)	Code	Control	T3	T8
Undecylenic acid	C11:1	3.00	-	-
9-Tetradecenoic acid	C14:1	0.35	-	-
Hexadecanoic acid (Palmitic acid)	C16:0	25.40	15.39	19.23
7-Hexadecenoic acid	C16:1	0.31	-	-
9-Hexadecenoic acid	C16:1	-	0.84	-
Hexadecenoic acid z-11	C16:1	0.31	-	-
9-Octadecenoic acid (oleic acid)	C18:1	59.46	44.51	38.65
cis-Vaccenic acid	C18:1	1.01	24.22	3.46
Ricinoleic acid	C18:1	8.86	-	-
cis-13-Octadecenoic acid	C18:1	-	-	7.88
trans-9-Octadecenoic acid (Elaidic acid)	C18:1	-	2.39	6.15
6-Octadecenoic acid	C18:1	-	3.58	-
trans-13-Octadecenoic acid	C18:1	1.29	5.25	14.81
11-octadecenoate	C18:1	-	-	3.85
cis-13-Eicosenoic acid	C20:1	-	-	2.69
9,12-Octadecadienoic acid (Z, Z)-	C18:2	-	3.82	3.27
% Saturated fatty acids		25.40	15.39	19.23
% Monounsaturated fatty acids		74.59	80.79	77.49
% Polyunsaturated fatty acids		00.00	3.82	3.27

For T1, the percentage of oleic acid in *T. obliquus* cells reached 59.46%, followed by palmitic acid (25.40%) and ricinoleic acid (8.86%). In contrast, at low nitrate conditions (T3), oleic acid constituted 44.51%, followed by cis-vaccenic acid (24.22%) and palmitic acid (15.39%). Additionally, under low sulfate conditions (T8), oleic acid comprised 38.65%, followed by palmitic acid (19.23%) and trans-13-octadecenoic acid (14.81%) (Table 5).

Based on the FAME profile, several biodiesel characteristics were calculated, with the results summarized in Table 6. The saponification value (SV), which indicates the amount of potassium hydroxide required to saponify one gram of oil, was 205.91, 187.92, and 201.69 mg KOH g^− 1^ fat for the control, T3, and T8, respectively. The iodine value (IV) for the treatments increased to 73.45 and 91.48 g I2 100 g^− 1^ fat in T3 and T8, respectively, compared to 68.23 g I2 100 g − 1 fat in T1. The estimated IV and cetane number (CN) values were within the limits established by various international standards (EN 14214, ASTM D6751, and IS 15607) (Table 6).

**Table 6:** Biodiesel characteristics of *T. obliquus* under under mixotrophic fed-batch treatments using 2 mL potato Peel hydrolysate incorporated every 2 days. Control: growth under nutrient sufficient medium, T3: low nitrogen (the AM lacked NaNO_3_ but contained 0.05 g L^–1^ of NH_4_Cl), T8: low sulphate (the AM contained 0.02 g L^–1^ of MgSO_4_.7H_2_O).

Biodiesel characters	Control	T3	T8	International Standards		
				EN 14,214	ASTM D6751-02	IS 15,607
**SV (mg KOH g**^**− 1**^ **fat)**	205.91	187.92	201.69	-	-	-
**IV (g I**_**2**_ **100 g**^**− 1**^ **fat)**	68.23	73.45	91.48	≤ 120	-	-
**CN**	57.45	58.82	52.78	≥ 51	≥ 47	≥ 51
**DU (wt%)**	74.60	88.42	84.04	-	-	-
**OS (h)**	4.91	4.37	4.54	≥ 6	≥ 3	≥ 6
**LCSF**	2.54	1.54	1.92	-	-	-
**CFPP (°C)**	−8.50	−11.64	−10.44	≤ 5/−20		6/18
**CP (°C)**	8.36	3.54	5.12	-	-	-
**PP (°C)**	2.26	−2.97	−1.26	-	-	3/15
**υ (mm**^**2**^ **s**^**− 1**^**)**	3.24	3.74	3.96	3.5 −5.0	1.9 −6.0	2.5 −6.0
***ρ*** **(g cm**^**− 3**^**)**	0.88	0.88	0.88	0.86–0.90	0.86–0.90	0.86–0.90
**HHV (MJ Kg** ^**− 1**^ **)**	39.29	39.82	39.97	-	-	-
**FP (°C)**	166.961	162.153	165.217	> 120	> 130	> 120

**EN**: European Committee for Standardization, **ASTM**: American Society for Testing and Materials, **IS**: Indian standard, **SV**: saponification value; **IV**: iodine value; **CN**: cetane number; **DU**: degree of unsaturation; **OS**: oxidation stability; **LCSF**: long chain saturation factor; **CFPP**: cold filter plugging point; **CP**: cloud point; **PP**: pour point; **υ**: kinematic viscosity; ***ρ***: density; **HHV**: higher heating value and **FP**: flash point.

The degree of unsaturation (DU) for the T8-derived biodiesel reached 84.04 wt%, while T3 exhibited a DU of 88.42 wt%, compared to 74.60 wt% for the control (Table 5). The estimated cold filter plugging point (CFPP) values were − 8.50, − 11.64, and − 10.44 °C for T1, T3, and T8, respectively. Moreover, T3 and T8 were characterized by lower cloud point (CP) and pour point (PP) compared to the control. The calculated viscosity (υ) and density (ρ) exhibited negligible variations between treatments, with values falling within the limits specified by international standards. Similarly, the higher heating value (HHV) and flash point (FP) values showed minimal differences across the treatments (Table 6).

## Discussion

The noteworthy positive impact of pH on the biomass productivity of *T. obliquus* suggests its alkaliphilic nature, demonstrating optimal growth within the pH range of 9–11. Raising the pH of the culture generally leads to an increased supply of bicarbonate and enhanced inorganic carbon uptake rates from the environment^16^. Furthermore, contaminating microorganisms and predators are suppressed under alkaline conditions (pH 11) which increases stability and minimizes culture crashes^16^. In the present study, a fed-batch mixotrophic cultivation method utilizing potato peel hydrolysate (PPH) was employed to enhance the biomass and lipid productivities of *T. obliquus* under alkaline conditions.

The method of hydrolysis of biomass has direct effects on its efficiency for mixotrophic growth of microalgae. Recent findings indicate that acidic hydrolysis of potato peels was ineffective in enhancing algal biomass production and can even inhibit growth due to the production of toxic by-products such as furfural and hydroxymethylfurfural^9^. Therefore, the potato waste was pretreated using fungal fermentation, a cost-effective and environmentally friendly process compared to acid hydrolysis, which relies on concentrated acids and high temperatures to produce fermentable sugars. Furthermore, fungal fermentation aligns with the principles of a circular economy, as it utilizes biological processes to valorize agricultural wastes. The duration of fungal pretreatment was optimized at 4 days to balance nutrient extraction and pretreatment efficiency, making it a time-efficient method for subsequent microalgae cultivation. The resulting PPH was rich in carbohydrates, with reducing sugars estimated at 2.18 g L⁻¹. The *T. obliquus* cells effectively utilized the PPH, as evidenced by increased biomass productivity compared to autotrophic conditions. These findings align with previous reports^8^, which indicated that fungal fermentation can depolymerize lignocellulosic waste into low molecular weight compounds readily utilized by microalgal cells. Incorporating 2 mL of PPH into the mixotrophic culture every two days significantly promoted algal biomass productivity. However, a higher PPH volume (3 mL) led to a decrease in *T. obliquus* growth compared to other treatments. This observation may be attributed to the detrimental effects of phenolic compound accumulation in the culture medium from elevated PPH volumes. Generally, PPH provided a direct organic carbon source, leading to faster cell division and higher biomass accumulation (1.8-fold compared to autotrophic growth). Furthermore, mixotrophic growth reduced reliance on light, enhanced energy efficiency, and boosted neutral lipid productivity (2.5-fold compared to autotrophic growth). This demonstrates that the integration of organic carbon supply, nutrient management, and alkaline cultivation provides a synergistic strategy for improving both biomass and lipid productivity.

In this study, mixotrophic cultivation was performed under alkaline conditions using a low concentration of potato peel hydrolysate in a fed-batch mode. This approach helps reduce microbial contamination and prevents culture crashes in large-scale production. The alkaline environment also supports favorable physiological stress conditions that can enhance lipid accumulation in many microalgal species while decreasing the need for sterilization, thereby lowering operational costs. Additionally, the simple fungal fermentation process utilized is more economically and environmentally friendly compared to chemical and enzymatic methods. The lipid productivity obtained in this study was comparatively higher than those reported for *Scenedesmus obliquus* cultivated using food wastewater^35^, and *Tetraselmis indica* using kinnow peel^36^, but lower than that reported for *Chlorella sorokiniana* using mixed peel extracts^37^. On the other hand, the composition of PPH in the present study (2.18 g L^− 1^ reducing sugars, 4.76 g L^− 1^ proteins, 0.18 g L^− 1^ lipids, and 0.04 g L^− 1^ phenolic compounds) is different from other biomass hydrolysates reported for mixotrophic microalgal cultivation. For example, fruit peel hydrolysates, such as orange peel, kinnow peel or mixed fruit wastes, often provide phenolics and organic acids along with sugars such as glucose and sucrose that can influence lipid metabolism and inhibit microalgal growth at elevated concentrations^36–38^. Wheat bran hydrolysate prepared by fungal fermentation contained higher reducing sugars but low protein contents, which favored biomass productivity but did not markedly promote lipid accumulation^8^. The moderate levels of sugars and minimal inhibitory phenolics in the PPH contributed to stable algal growth and elevated lipid productivity. These differences highlight that hydrolysate composition plays a crucial role in determining microalgal metabolic responses, and further comparative studies are required.

Variations in nutrient concentrations within algal cultures play a crucial role in influencing biomass and lipid productivity. Nitrogen limitation or deprivation has been reported to induce the hyperaccumulation of cellular lipids, although it negatively affects biomass production^39,40^. Consistent with these findings, the present study indicated a decline in biomass productivity of *T. obliquus* under nitrogen-deficient conditions compared to the control. Accordingly, nitrate was identified as the most important factor for promoting the biomass productivity of *T. obliquus* under fed-batch mixotrophic growth using PPH. However, the balance between inorganic nutrients, mainly nitrate, phosphate, and sulphate, and external organic carbon (PPH) is crucial to maintain microalgal growth and biomass production. The improved biomass productivity of *T. obliquus* at low or deprived phosphate or sulphate during mixotrophic fed-batch cultivation may indicate that nitrogen availability could promote the rate of sugar uptake from PPH and biomass production under phosphate- or sulfate-deprived conditions. This observation was supported by the results of Phalanisong et al. who reported an increase of cumulative sugar consumption from sugarcane juice and increased biomass production of microalgae consortia under P-limited conditions compared to N-limited conditions^12^.

Interestingly, under low initial nitrogen concentration (T3, 0.05 g L⁻¹ of NH₄Cl), the algal cells accumulated more neutral lipids, resulting in a notable increase in lipid productivity, approximately 1.25-fold. Similarly, Gao et al. reported a simultaneous increase in lipid content and a reduction in biomass productivity, achieving an overall 1.1-fold increase in the lipid productivity of *Parachlorella kessleri* cultivated mixotrophically under nitrogen-deficient conditions^41^. High pH and nutrient-limited conditions have also been shown to promote the accumulation of neutral lipids in microalgae^42^. Generally, under nitrogen deficiency, microalgae tend to degrade nitrogen-containing cellular compounds, leading to an increased storage of lipids and carbohydrates^43^.

Under fed-batch mixotrophic conditions, decreasing phosphate concentrations resulted in lower levels of neutral lipids (NL) and glycolipids (GL), while promoting the accumulation of phospholipids (PhL) compared to the control. Microalgae can accumulate phosphorus (P) from the culture medium under P-replete conditions, storing it as polyphosphate granules for reuse during periods of P starvation^44^. However, microalgal cells require a significant amount of time to adapt to phosphate-deprived conditions before entering the logarithmic phase of rapid cell division, as evidenced by a notable increase in lag time under phosphate deprivation relative to nutrient-sufficient treatments. Previous studies have reported that under phosphorus deficiency, PhL are generally replaced by non-phosphorus GL, leading to increased levels of NL as an effective P-conserving mechanism^45,46^. The present study observed a significant negative correlation between the contents of PhL and GL (Pearson’s *R* = − 0.90, *P* = 0.001), consistent with findings from earlier research^39,47^. In phosphate-replete conditions (T1), the contents of PhL decreased while GL increased; conversely, the opposite trend was observed under phosphate-deprived (T5) or limited (T6) conditions. This behavior may reflect the metabolic adjustments microalgae undergo to cope with nutrient limitations. Overall, the response of microalgae to P limitation is species-specific. For some species, such as *Phaeodactylum tricornutum*, *Chaetoceros* sp., and *Pavlova lutheri*, P limitation induces lipid accumulation^48^. In contrast, species such as *Nannochloris atomus*, *Tetraselmis* sp., *Chlorella*^48,49^, and *Botryococcus sudeticus*, along with *C. sorokiniana* and *T. suecica*^50^, experience decreased lipid contents. This reduction may be due to the accumulation of carbohydrates rather than NL^48,50^.

Similarly, sulfate deficiency increased the contents of phospholipids (PhL) at the expense of glycolipids (GL), while the levels of neutral lipids (NL) showed non-significant changes compared to the control. The effects of sulfate concentration on lipid accumulation in microalgae are generally species-specific, and their impact can vary between non-significant, positive, and negative effects^50^. Furthermore, sulfur (S) deprivation has been reported to upregulate genes associated with sulfolipid biosynthesis, which can be hydrolyzed to provide a source of S for cellular metabolic activities^51,52^.

Maintaining PhL under nutrient deficiency is crucial for the structural integrity and functionality of chloroplasts, as these compounds are integral components of thylakoid membranes and play a fundamental role in the activity of photosystems I and II^53^. Thus, sustaining the photosynthetic efficiency of *T. obliquus* cells provides sufficient carbon for cell division and growth. Consequently, maximum biomass productivity (~ 1.2-fold higher than the nutrient-sufficient control) was observed under phosphorus (P) and sulfur (S) deficient or deprived conditions. In a related study, Sakarika and Kornaros reported a significant increase in the biomass productivity of *Chlorella vulgaris* under P-limited heterotrophic conditions^54^. However, the same strain exhibited a substantial reduction in biomass productivity under S limitation, attributed to its higher demand for S to produce sulfur-containing compounds compared to other microalgae^54^.

Generally, nitrogen (N) starvation has a more immediate and adverse effect on cell division compared to phosphorus due to the presence of stored P in the form of polyphosphates^55^. The present results similarly indicated a significant reduction in algal biomass productivity under nitrogen starvation compared to P- or S-deprived conditions. This behavior may also stem from lower consumption rates of organic carbon under N-deficient mixotrophy compared to P-limited conditions^56^. Furthermore, the presence of organic carbon from potato peel hydrolysate (PPH) in the culture medium enabled *T. obliquus* to utilize mixotrophic energetic metabolism under nutrient-limited conditions, supporting its requirements for cellular division.

Lipid productivity, which is derived from biomass productivity and cellular lipid content, is a fundamental indicator of oil-producing capacity. Under fed-batch mixotrophic cultivation, the highest neutral lipid productivity was achieved at low nitrogen conditions (T3: 20.90 mg L⁻¹ day⁻¹), low sulfate (T8: 22.61 mg L⁻¹ day⁻¹), and sulfate-deprived conditions (T7: 20.77 mg L⁻¹ day⁻¹). Previous studies have indicated that phosphorus plays a crucial role in enhancing lipid productivity under nitrogen-deficient conditions, facilitating the production of energy transfer molecules and nucleic acids^49^.

Saturated fatty acids (SFAs) and monounsaturated fatty acids (MUFAs) generally exist as neutral lipids, which are essential for biodiesel production, while polyunsaturated fatty acids (PUFAs) are typically found in polar lipids. Therefore, high-quality biodiesel should contain long-chain fatty acids with a low level of unsaturation. The biodiesel obtained from *T. obliquus* in this study was characterized by a higher percentage of MUFAs compared to SFAs, with very little PUFA present. Moreover, all fatty acids detected were either C16 or C18. Similarly, a recent study indicated that 10% v/v PPH in BG-11 medium of *Spirulina* sp. induced higher levels of MUFAs (C18:1 and C16:1) than SFAs and PUFA^11^. Additionally, previous studies have shown that increasing CO₂ concentration in the culture medium promotes the production of C18:1 over C16:1, while adversely affecting the production of C18:2, C16:0, and C18:3^57^.

Ideally, biodiesel should contain lower quantities of PUFAs and SFAs compared to MUFAs to mitigate issues related to oxidative stability and cold flow^6,58,59^. The biodiesel obtained from the optimized treatments exhibited a higher percentage of monounsaturated fatty acids (MUFAs), which could improve biodiesel oxidative stability and cold flow properties. Furthermore, low PUFA minimizes polymerization risks. The cetane number (CN), which relates to the ignition quality of fuel in diesel engines, was found to be elevated in the investigated treatments, ranging between 52.78 and 58.82. These values exceed the minimum required by international standards, which is 51, and are associated with a higher concentration of saturated and monounsaturated methyl esters. Enhanced CN levels contribute to superior combustion, improving engine efficiency and reducing nitrogen oxides emissions^60^.

Overall, the integration of alkaline cultivation with PPH and controlled nutrient limitation demonstrated a synergistic effect that enhanced both biomass and neutral lipid productivity. From economic perspective, the utilization of zero-cost potato waste can substantially reduce carbon source expenses in mixotrophy compared to glucose. The major costs in scaling-up would be associated with the photobioreactor design, along with the energy requirements for aeration, mixing, and downstream processing such as harvesting, and lipid extraction. Therefore, further optimization in photobioreactor or open pond systems would be essential to validate the scalability of this approach.

## Conclusion

This study evaluated the growth of *T. obliquus* under extreme alkaline conditions (pH 11) using potato peel hydrolysate (PPH) as a sustainable and low-cost source of organic carbon. The fed-batch mixotrophic cultivation significantly promoted the biomass and lipid productivity of the microalga. Notably, the productivity of non-polar lipids experienced remarkable enhancements under low nitrate, low sulfate, and sulfate-deprived conditions, reaching 20.90, 22.61, and 20.78 mg L⁻¹ day⁻¹, respectively, with the addition of 2 mL of PPH every 2 days. These values were approximately three-fold higher than the autotrophic control level of 7.44 mg L⁻¹ day⁻¹. Furthermore, the biodiesel produced under the optimized mixotrophic conditions was rich in C16 and C18 fatty acids, exhibiting characteristics that align with international specifications. These findings highlight the potential of cultivating microalgae under extreme alkaline mixotrophic conditions for biodiesel production. However, large-scale production requires optimized bioreactor designs with high pH resistance, aeration strategies, and cost-effective harvesting methods.

## Data Availability

The datasets used and/or analyzed during the current study are available from the corresponding author on reasonable request.
